# The Crystal Structure of the R280K Mutant of Human p53 Explains the Loss of DNA Binding

**DOI:** 10.3390/ijms19041184

**Published:** 2018-04-13

**Authors:** Ana Sara Gomes, Filipa Trovão, Benedita Andrade Pinheiro, Filipe Freire, Sara Gomes, Carla Oliveira, Lucília Domingues, Maria João Romão, Lucília Saraiva, Ana Luísa Carvalho

**Affiliations:** 1 LAQV-REQUIMTE, Laboratório de Microbiologia, Departamento de Ciências Biológicas, Faculdade de Farmácia, Universidade do Porto, 4050-313 Porto, Portugal; anasarag4@gmail.com (A.S.G.); up200802730@icbas.up.pt (S.G.); 2 UCIBIO-REQUIMTE, Departamento de Química, Faculdade de Ciências e Tecnologia, Universidade Nova de Lisboa, 2829-516 Caparica, Portugal; f.trovao@campus.fct.unl.pt (F.T.); b.pinheiro@fct.unl.pt (B.A.P.); f.freire@campus.fct.unl.pt (F.F.); maria.romao@fct.unl.pt (M.J.R.); 3 CEB—Centre of Biological Engineering, University of Minho, Campus Gualtar, 4710-057 Braga, Portugal; carlaoliveira@deb.uminho.pt (C.O.); luciliad@deb.uminho.pt (L.D.)

**Keywords:** mutant p53R280K, crystal structure, DNA binding, anticancer therapy

## Abstract

The p53 tumor suppressor is widely found to be mutated in human cancer. This protein is regarded as a molecular hub regulating different cell responses, namely cell death. Compelling data have demonstrated that the impairment of p53 activity correlates with tumor development and maintenance. For these reasons, the reactivation of p53 function is regarded as a promising strategy to halt cancer. In the present work, the recombinant mutant p53R280K DNA binding domain (DBD) was produced for the first time, and its crystal structure was determined in the absence of DNA to a resolution of 2.0 Å. The solved structure contains four molecules in the asymmetric unit, four zinc(II) ions, and 336 water molecules. The structure was compared with the wild-type p53 DBD structure, isolated and in complex with DNA. These comparisons contributed to a deeper understanding of the mutant p53R280K structure, as well as the loss of DNA binding related to halted transcriptional activity. The structural information derived may also contribute to the rational design of mutant p53 reactivating molecules with potential application in cancer treatment.

## 1. Introduction

The p53 tumor suppressor protein is a central regulator of cell proliferation, DNA repair, differentiation, and death. The loss of p53 transcriptional activity may result in uncontrolled cell proliferation and in the accumulation of genomic injuries that culminate in tumor growth. In fact, the impairment of p53 has long been recognized as a signature of human cancer [1,2].

Structurally, the p53 protein comprises constitutes an intrinsically disordered N-terminal transactivation domain (TAD), a folded core domain or DNA binding domain (DBD), and a C-terminal oligomerization domain (OD). In its folded form, the DBD exhibits a β-sandwich topology with two loops and a loop-sheet-helix motif. The structural zinc(II) ion is coordinated by four amino acid residues, namely C176, H179 (in loop L2) and C238, C242 (in loop L3), ensuring the correct folding of the DBD through the stabilization of these two loops. This stabilization of L2-L3 by zinc(II) plays an important role in p53 transcriptional activity once it allows a correct positioning of residue R248 that interacts with DNA’s minor groove [3]. The OD allows p53 monomers to ensemble as a tetramer (dimer of dimers) crucial for its transcriptional activity. In this active conformation, p53 cooperatively binds to specific DNA sequences called response elements (RE) present in the promoter region of target genes. DNA recognition is held by the loop-sheet-helix motif in the DBD, specifically by K120, S241, R273, A276, R283, C277, R248, and R280 residues, and by the TAD. Additionally, residues R175, G245, R249, and R282 are also important for correct folding and DNA-p53 complex stability [4,5].

In over half of all human cancers, p53 is mutated [1]. Most p53 mutations are missense preferentially localized in its DBD. The DBD is highly affected by these mutations due to the dynamics of p53 folded and intrinsically disordered domains, which influences specificity in DNA binding, and due to its low intrinsic thermodynamic stability [6,7]. Such events result in the impairment of DNA transcriptional activity, leading to mutant p53 loss of function. Even in tumors that still bear one allele for wild-type (wt) p53, the mutant form can exert a dominant negative effect over wt p53. Additionally, it has been reported that some mutant p53 proteins may acquire new oncogenic properties, called gain of function (GOF), through the recognition of genes related to oncogenesis or by hetero-oligomerization with p63/p73 and other transcription factors [8,9,10]. p53 mutants can be classified as contact mutants (i.e., R248Q, R273H, and R280K) if the mutation occurs in a residue directly involved in DNA binding without causing protein unfolding, or as structural mutants (i.e., Y220C, R249S, and R282W) if the mutation induces a destabilization leading to structural distortion, unfolding, or aggregation [7].

The zinc(II) ion was found to regulate the transcriptional activity of wt p53 and zinc(II)-deficient mutants, such as structural mutant R175H. It was also observed that zinc(II) chelation halted wt p53 transcriptional activity by inducing its structure unfolding, an effect reverted after zinc(II) supplementation [11,12]. Additionally, it was shown that mutant p53R175H loss of function can be rescued after treatment with zinc(II)-complexes [13,14]. However, there are also other structural mutant forms of p53 that are not zinc(II)-deficient, such as p53Y220C and p53R282W, whose loss of function is related to the thermodynamic destabilization of their structures [6]. Also, for contact p53 mutants such as p53R273H, p53R273C, and p53R248W, their loss of function has been directly associated with an impairment of DNA binding due to the loss of the arginine’s guanidinium group [3].

Mutant p53 has high clinical relevance, with expressive prevalence (25–45%) in colorectum, head and neck, esophagus, female genital organs, and urinary tract cancers (IARC (International Agency for Research on Cancer) TP53 Database, R18, April 2016, [15]). Moreover, it is usually associated with tumorigenesis, tumor progression, more resistant and invasive tumor phenotypes, and thereafter with poor prognosis [16,17]. As such, many efforts have been focused on targeting p53 as a therapeutic strategy to halt cancer. Indeed, to date, several new chemical entities have been reported with the ability to restore wt-like function to mutant p53, with PRIMA-1^MET^, COTI-2, PEITC, WR-1065, and aminoglycosides reaching clinical trials [18,19,20]. Nevertheless, p53 structural studies and a full understanding of this protein in a biological context are challenged by its structural dynamics [21,22,23]. The structure of the p53 DBD in complex with DNA was first reported in 1994 [4]. After that, along with the evolution of structural analysis techniques, many other structure models have been reported regarding the full-length protein or structural elements of p53 and its mutants [24,25]. So far, different mutated p53 forms with clinical impact have been studied regarding their thermodynamic stability and DNA binding, some of which were structurally elucidated (reviewed in Reference [26], IARC *TP53* Database, p53 thermodynamic stability, [15]). These studies have contributed to the understanding of the biology/biochemistry of this molecular hub, and to the rational design of p53-reactivating agents. In particular, the elucidation of the mutant p53Y220C structure has enabled a rational in silico drug design that led to the discovery of a class of molecules with a carbazole scaffold able to restore the wt-like folding to this mutant form [27].

The present work focuses on the mutation of residue R280, which is involved in direct DNA recognition and interacts with a structural water molecule [7,28]. When this residue is mutated (R280K), the DNA transcription is halted [29]. This contact mutant has also been described to acquire GOF with clinical relevance, related to the transcription of noncanonical p53 target genes, namely *MAP2K3* (involved in increased cell proliferation), *MVK* and *FDFT1* (involved in lipid metabolism with tumor progression), *CENP-A* (related to chromosomal instability), and the more recently reported *ENTPD5* (promoting cell proliferation and colonization) [8,18,30,31]. Mutant p53R280K has been related to different cancer types, such as ureter, gallbladder, bladder, and prostate cancers (IARC *TP53* Database, R18, April 2016, [15]). Herein, the structure model of the recombinant human mutant p53R280K DBD (without the four stabilizing mutations described in Reference [22]) was obtained by X-ray crystallography for the first time. A comparison is made with previously reported model structures of wt p53 DBD, isolated and in complex with DNA.

## 2. Results

### 2.1. Expression and Purification of Mutant p53R280K DBD

After expression in BL21 (DE3) *Escherichia coli* cells, the DBD of the mutant p53R280K (residues 94–312) was purified by cation exchange followed by heparin-affinity chromatography, as described by Bullock and colleagues [32]. However, the produced protein was shown to be not suitable for crystallographic assays since it was very unstable, presenting aggregation after 24 h. By differential scanning fluorimetry (DSF) and dynamic light scattering (DLS) assays, performed with protein samples after each step of purification, it was possible to infer that after the cation exchange chromatography the protein was unfolded. In fact, in DSF, a high interaction with SYPRO^®^Orange was detected through high relative fluorescence units (RFU) at low temperatures, indicating that the protein is already unfolded at the starting point. Furthermore, protein size analysis through DLS revealed that the protein was also aggregating after cation exchange chromatography. Nevertheless, heparin-affinity chromatography rescued mutant p53R280K DBD folding, showing a size population around 6 nm (in accordance with its molecular weight), probably due to the affinity of DBD to heparin [33]. These results led us to consider that a smoother purification process should be adopted in this specific case, and an optimization of buffer should be pursued. As such, the purification was optimized by performing only heparin-affinity chromatography, followed by gel filtration (in Supplementary Figure S1). Additionally, to improve protein stability, DSF screening of different buffers and additives was performed to select the best buffer (in Supplementary Figure S2). It was also verified that buffer supplementation with zinc(II) ion, which is a required factor for the correct folding of p53 DBD [34], contributes to protein stability. Considering the thermal stabilization of mutant p53R280K DBD, the selected buffer (SB) was 50 mM HEPES pH 7.5, 150 mM NaCl, 5 mM DTT, 10 µM Zn(CH_3_COO)_2_, and 5% (*v*/*v*) glycerol. Altogether, a purification protocol was optimized to produce a mutant p53 suitable for biophysical assays, particularly crystallography.

### 2.2. Crystallization and Structural Elucidation of Mutant p53R280K DBD

**Figure 1:**
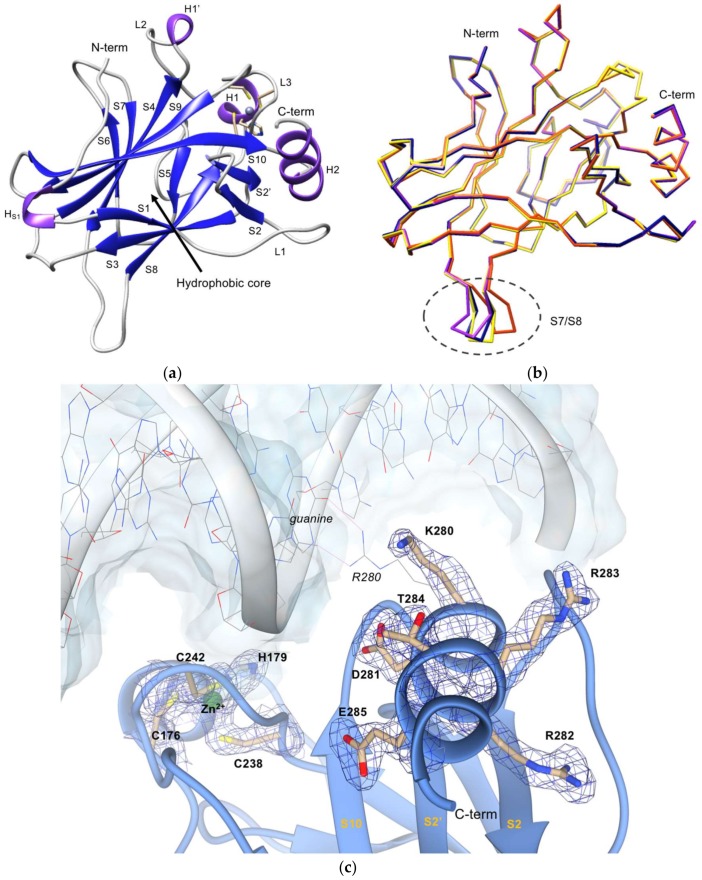
Crystal structure of p53R280K DBD. (**a**) Ribbon diagram of p53R280K DBD; strands are shown in dark blue, helices in purple, linked by gray coils. The zinc(II) ion is represented by a gray sphere near L3 loop and H1 helix and its coordinating amino acid residues (C176, H179, C238, C242) are shown as yellow sticks. (**b**) Backbone superposition of the four molecules of p53R280K DBD in the asymmetric unit of the crystals. Molecule A, purple; molecule B, yellow; molecule C, blue; molecule D, orange. Encircled by a gray dashed line is the region with the highest structural variation, the S7/S8 turn. (**c**) Electron density map calculated around the C-terminus residues and the zinc(II) ion, in green (2mF_o_-DF_c_ map at 1σ level and 2 Å resolution). The p53R280K DBD polypeptide chain is represented in blue ribbon. Clear electron density for the lysine residue at position 280 is seen in all molecules of the asymmetric unit in the p53R280K DBD domain structure. The DNA fragment and the arginine residue of the wt form (PDB code 2AC0) are superposed to illustrate the orientation of the protein in relation to the DNA and are depicted in color-coded wire-frame and labeled in italic. The two direct hydrogen contacts with guanine, that are disrupted in the p53R280K structure, are depicted as pink thin lines. Residues from p53R280K are labeled in bold, while residues in the wt complex are labeled in italics.

After recombinant mutant p53R280K DBD production and concentration, the pure protein was crystallized using polyethylene glycol (PEG) 3350 as a precipitant. Crystals grew in space group *P*2_1_, which contains four molecules in the asymmetric unit and each molecule comprises amino acid residues 97 to 290. Coordinates and observed structure factor amplitudes for the human p53R280K mutant, to 2.0 Å resolution, have been deposited in the Protein Data Bank in Europe (PDB ID code 6FF9). The overall fold of the p53R280K structure is similar to structure of the wt p53 DBD in the absence of DNA [35] (Figure 1). Like other p53 structures already reported, the p53R280K DBD presents an immunoglobulin-like β-sandwich fold with two twisted antiparallel β-sheets of four (S1, S3, S4, and S5) and five (S10, S9, S4, S7, and S6) strands, respectively, forming a hydrophobic inner core (Figure 1a). The structure also contains a series of loops (L1, L2, and L3) at opposite ends of the β-sandwich and two short helices (H1 and H2) at one of the two ends. The two large loops (L2 and L3) and a loop-sheet-helix motif compose the DNA binding surface. As observed in wt p53 and in some mutant p53 forms [4,6,36,37,38], in mutant p53R280K, the L2 and L3 loops are stabilized by a zinc(II) ion, which is tetrahedrally-coordinated to C176, H179, C238, and C242 (Figure 1c). The loop-sheet-helix motif contains L1 loop, the S2-S2′ hairpin, and the C-terminal residues of the extended β-strand S10, as well as the H2 helix. In this structure, it was also possible to observe two extra short helices (H_S1_ and H1′), as observed in other p53 mutants [37,39].

The superposition of the four molecules of p53R280K DBD present in the asymmetric unit shows root-mean-square deviation (r.m.s.d.) values of 0.40 Å, 0.32 Å, and 0.35 Å for 193 backbone atoms (molecule A superposed on molecules B, C, and D, respectively) (Figure 1b). Contrary to the DNA-binding interface, the turn between S7 and S8 strands (residues 220–229), stands out as the region with the major structural variation. As reported in other p53 DBD structures, this region shows an inherent flexibility [35,40,41]. The maximal distance observed between C^α^ atoms of V225 is 6.19 Å. Additionally, at 2.0 Å resolution, it was also possible to confirm the mutation of p53R280K DBD at position 280, as the density for K280 was clear in all four molecules present in the asymmetric unit (Figure 1c). This K280 is located at the H2 helix, which belongs to the loop-sheet-helix motif of the DNA binding surface. In the wt DNA-bound p53, the R280 residue is directly involved in DNA recognition, in addition to interacting with a structural water molecule [7].

## 3. Discussion

The studied contact mutation R280K disables the binding of p53 to DNA, halting DNA transcription as previously reported [29]. To further understand the effect of R280K mutation on DNA binding, the mutant p53R280K DBD structure is here compared with DNA-free and DNA-bound wt p53 DBD.

**Figure 2:**
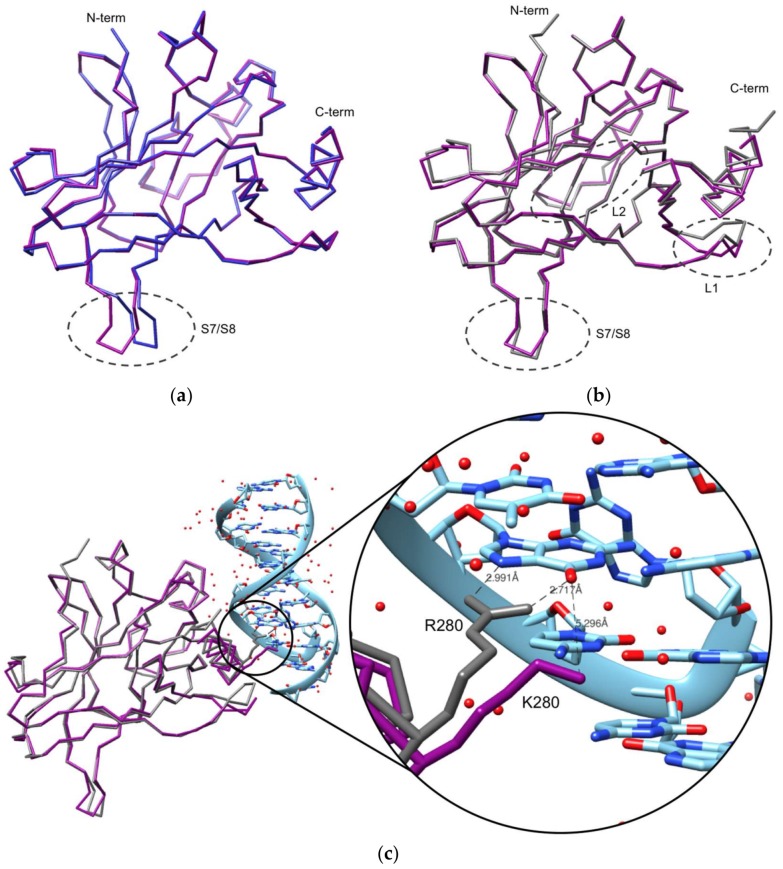
Comparison of p53R280K DBD (backbone in purple) with (**a**) human DNA-free (2OCJ, blue) and (**b**) DNA-bound (2AC0, gray) wt p53 DBD structures. Regions with the highest structural variations are encircled by gray dashed lines. In (**a**), the S7/S8 turn is the region with the highest structural variation, while in (**b**), the S7/S8 turn, L1 loop, and L2 loop are the regions with the most significant structural differences. The L2 loop is located at the back in this figure. (**c**) Backbone superposition of p53R280K DBD (purple) with the p53 DBD in complex with DNA (2AC0, gray). The double-stranded DNA is represented in cyan blue. The side chains at position 280 are shown in both structures, lysine from mutant p53 and arginine from wt p53. The hydrogen contacts between the R280 and guanine, and a hypothetical contact between K280 and guanine, are represented by gray dashed lines; carbon atoms are colored light blue, nitrogens are navy blue and oxygens are red. Atomic distances are indicated.

The superposition of the p53R280K DBD monomer structure with the DNA-free wt p53 DBD structure (PDB ID: 2OCJ, [41]) results in average r.m.s.d. values of 0.3 Å (for 193 aligned residues) among the independent monomers. These r.m.s.d. values are similar to those found in the independent superposition of each of the four molecules of the p53R280K DBD. Also, no relevant structural changes are found between monomers, besides the S7/S8 turn (inherent flexibility) (Figure 2a). Furthermore, the R280K mutation does not affect the overall p53 structural conformation, harboring one zinc(II) ion coordinated to four amino acid residues. As seen in other crystal structures of the unbound p53, although four molecules are commonly found in the asymmetric unit, their packing is quite diverse and does not reflect the dimer of dimers arrangement observed in the B-DNA (decameric half sites)-bound complex [28]. Still, when superposed with the DNA-free wt p53 structure, the dimer of mutant p53 matches the dimer of wt p53 (with an r.m.s.d. of 0.3 Å for 386 aligned residues). Also, the contacts in these dimers show similar interfaces in the two structures, forming hydrogen bonds and salt bridges, involving the H_S1_ helix (molecule A) with the L3 loop and H1 helix (molecule B), and the S9/S10 turn (molecule A) with the H1 helix (molecule B). The interaction between dimers is also stabilized by hydrogen bonds and salt bridges, which involve the L1 loop with the S6 strand, and the H2 helix with the S9/S10 strand and S5/S6 strand.

When comparing the p53R280K DBD structure with the DNA-bound wt p53 (PDB: 2AC0, [28]), slightly higher r.m.s.d. values (between 0.66 and 0.71 Å) are observed. The overall structure of the p53R280K DBD monomer is also similar to that of the wt p53 monomer in complex with DNA; however, some significant structural differences can be found in certain regions, such as the S7/S8 turn, L1 loop, and L2 loop (Figure 2b). The variations observed in the S7/S8 turn are due to the inherent flexibility of this region. The L1 and L2 loops variations result from the absence of DNA. In the L1 loop, K120 and S121 have the largest deviations, and the C^α^ atoms are displaced by 2.44 and 4.14 Å, respectively. It is already known that K120 contributes to DNA binding with two hydrogen bonds, so when bound to DNA, the L1 loop needs to rearrange and fit into the major groove of the DNA molecule [41]. Another structural difference is observed in the L2 loop near the DNA binding-surface (C182-G187), where the C^α^ atoms of S183 and G187 are displaced by 1.60 and 1.15 Å, respectively.

Most relevant to this study are the structural differences observed at the mutation site (R280). In the DNA-bound wt p53 structure, the guanidinium group of R280 forms two hydrogen bonds with a guanine nucleotide of consensus DNA, while K280 (in p53R280K) would only be capable of forming one hydrogen bond with DNA. Superposition of both monomer structures clearly shows that the distance between K280 (from p53R280K) and the DNA molecule is longer, a consequence of the shorter side chain of lysine compared to arginine (Figure 2c), as also observed in other contact mutants p53R273H and p53R273C [37]. Associated with this is the known fact that arginine presents a more positively-charged side chain compared to lysine, which favors electrostatic interaction with the negatively-charged DNA molecule. Due to the increased distance and weaker binding, p53R280K is unable to form stabilizing interactions with DNA, which consequently causes its loss of function, despite other well-established contacts that could be maintained. Furthermore, the replacement of an arginine residue by lysine can significantly affect a protein’s stability [42] and, in the case of p53, its DNA binding efficiency.

## 4. Materials and Methods

### 4.1. Expression Plasmid Construction of Mutant p53R280K DBD

The DBD of the mutant p53R280K (residues 94–312) was PCR-amplified from vector pLS76 [43] with Vent DNA polymerase (New England Biolabs, Ipswich, MA, USA), using the forward primer 5′ TGC **TCTAGA** AAT AAT TTT GTT TAA CTT TAA GAA GGA GAT ATA CAT *ATG* TCA TCT TCT GTC CCT TCC 3′ and the reverse primer 5′ CCG **CTCGAG** TCA GGT GTT GTT GGG CAG 3′. The restriction sites *Xba*I and *Xho*I (in bold) were included in the forward and reverse primers, respectively, and a start codon (italics) and a stop codon (underlined) were also respectively included in the forward and reverse primers. PCR products were digested with *Xba*I and *Xho*I (New England Biolabs) and inserted into the same restriction sites of the pETM20 expression vector (EMBL, Heidelberg, Germany). For that, digested DNA fragments were gel purified using the Qiaquick Gel Extraction Kit (QIAgen, Hilden, Germany) and ligated with T4 DNA ligase (Promega, Madison, WI, USA), according to the manufacturer’s protocols, originating the plasmid pETM20-p53R280K. This plasmid was propagated and maintained in *Escherichia coli* NZ5α (NZYTech, Lisboa, Portugal). The sequence of the insert in the plasmid was confirmed by sequencing (GATC Biotech, Constance, Germany) with T7 primers pair. The expression plasmid pETM20-p53R280K was then transformed into the *Escherichia coli* expression strain NZYBL21 (DE3) (NZYTech).

### 4.2. Recombinant Production and Purification of Mutant p53R280K DBD

Protein production and purification was based on the methods of Reference [32], with further optimization. *Escherichia coli* BL21 (DE3) cells, harboring the recombinant plasmid pETM20-p53R280K, were grown at 37 °C (310 K) in LB (Luria-Bertani broth) medium supplemented with 100 µg/mL of ampicillin to an OD_600 nm_ of 1.2. The bacterial culture was thereafter supplemented with 10 µM of Zn(CH_3_COO)_2_, and recombinant protein expression induced with 1 mM IPTG (isopropyl β-d-thiogalactoside) overnight at 25 °C. Afterward, cells were recovered by centrifugation and lysed in 50 mM HEPES (4-(2-hydroxyethyl)-1-piperazineethanesulfonic acid) pH 7.5, 5 mM dithiothreitol (DTT), 10 µM Zn(CH_3_COO)_2_, and 100 mM phenylmethylsulfonyl fluoride (PMSF) using sonication. Cellular remains were removed by centrifugation. Filtrated (0.2 µm) supernatant was loaded onto a HiTrap™ Heparin HP column (GE Healthcare, Little Chalfont, Buckinghamshire, UK) and proteins eluted with a NaCl step gradient (0, 100, 200, and 250 mM, 1 mL/min, and 0.7 mL/min for the last concentration), in ÄKTA start (GE Healthcare). The eluted fractions, containing the p53R280K, were pooled and further purified by gel filtration chromatography using a Superdex-75 column (GE Healthcare) in a Shimadzu HPLC (0.5 mL/min) and the selected buffer (SB) with 50 mM HEPES pH 7.5, 150 mM NaCl, 5 mM DTT, 10 µM Zn(CH_3_COO)_2_, and 5% (*v*/*v*) glycerol as the running buffer. Peak fractions were analyzed by SDS-polyacrylamide gel electrophoresis (Blue Coomassie staining) and fractions containing pure protein (~25 kDa) were combined and concentrated using an Amicon Ultra-15 Centrifugal Filter Unit (Cut-off: 10 kDa; Cycles: 3500 g, 10 min, 4 °C) for crystallization assays.

### 4.3. Differential Scanning Fluorimetry (DSF) Screening for Buffer Optimization

A protocol was adapted from Reference [44]. Briefly, two screenings were made, one for buffers/pH and another for additives. Ninety-six-well plates were used (MicroAmp^®^ Fast 96-well Reaction Plate (0.1 mL) from Applied Biosystems, Foster City, CA, USA) and were placed on ice. A reaction mixture of 20 µL was prepared in each well: 11 µL of the buffer/additive solution to be screened, 2 µL at 15 µM of mutant p53R280K DBD (final concentration of 1.5 µM), and 7 µL of SYPRO^®^Orange dye (5×; prepared in Protein Thermal Shift Dye Kit™, from Applied Biosystems) was added and mixed. The plate was sealed, centrifuged (1 min, 200*g*, 4 °C) to remove air bubbles, and placed on ice for 5 min in a dark place to equilibrate. DSF was performed using a StepOnePlus Real-Time PCR system (Applied Biosystems) with ROX (rhodamine X; 575/602 nm, absorption/emission) filters. The temperature scan was performed using the range from 25 to 95 °C, at 1 °C/min. Data was exported and melting temperatures (Tm) were analyzed in Microsoft Office Excel.

### 4.4. Crystallization of p53 R280K DBD

Crystallization screens were performed using an automated crystallization robot (Oryx8, Douglas Instruments, Hungerford, UK) on 96-wells plates, where 0.67 µL of protein solution (6.6 mg/mL in SB) were mixed with 0.33 µL reservoir buffer (1 µL drops with a proportion of 2:1). The best crystals were grown at 20 °C (293 K) using a sitting drop vapor diffusion technique against a reservoir containing 35% (*w*/*v*) polyethylene glycol (PEG) 3350. Colorless plate-shaped crystals appeared within a week and continued to grow for one more week (in Supplementary Figures S3a,b). Crystals were stabilized in the harvest buffer (38% (*w*/*v*) PEG 3350) and flash-frozen in cryoprotectant buffer (38% (*w*/*v*) PEG 3350, 10% glycerol) with liquid nitrogen. 

### 4.5. Data Collection, Structure Solution, and Refinement

X-ray diffraction data from crystals of p53 R280K DBD were collected at the ID30A-3 beamline of the European Synchrotron Radiation Facility (Grenoble, France), to a maximum resolution of 2.0 Å, using an energy of 12.81 keV (in Supplementary Figure S3c), a crystal-to-detector distance of 144.8 mm, and an oscillation angle of 0.15°. The diffraction data were indexed in *P*2_1_, and integrated and scaled using the CCP4 software package [45]. The structure was solved by molecular replacement using *PhaserMR* [46], with a wt p53 DBD (Protein Data Bank code 2OCJ) as the search model [41]. All subsequent refinement cycles were carried out in the *Phenix* platform [47], using the program phenix.refine [48] with the web server *PDB-REDO* [49]. All residues had backbone and angles in the allowed region of the Ramachandran plot, with 99.7% in the favored region. The resulting *R*_work_ and *R*_free_ were 0.20 and 0.24, respectively. All data collection and refinement statistics are summarized in Table 1. Figures were generated with UCSF Chimera [50] and PyMOL [51].

## 5. Conclusions

In conclusion, the described crystal structure of mutant p53R280K provides a deeper understanding about this mutant p53 structure as well as its impact on DNA binding. In fact, the mutant p53R280K DBD crystal structure revealed that, as in wt p53, there is a correct folding of mutant p53R280K DBD, without any crevice and with zinc(II) ion coordination. Most importantly, it indicated that the loss of function is likely related to the inability of the residue K280 to establish two hydrogen bonds with DNA. This knowledge may help in unravelling the biology and activity of mutant p53. Additionally, it may contribute to the rational design of new targeted anticancer therapies. In fact, candidate drugs for the reactivation of mutant p53R280K function could, for example, fill in the cavity created by the absence of the arginine side chain, restoring the missing hydrogen contacts and/or strengthening the electrostatic interaction.

## Figures and Tables

**Table 1:** X-ray diffraction, model building, and refinement statistics (values for the outer shell are given in parentheses).

Data Collection and Processing	
X-ray source	ESRF, ID30B
Wavelength (Å)	0.9677
Space group	*P* 1 2_1_ 1
Unit-cell parameters (Å, °)	*a* = 68.6, *b* = 69.4, *c* = 83.3, β = 90.04
Resolution range (Å)	41.67–2.0 (2.07–2.0)
Solvent content (%)	39
Protein molecules per asymmetric unit	4
Matthews coefficient (Å^3^.Da^−1^)	2.01
Mosaicity (°)	0.33
*I*/*σ (I)*	8.6 (2.0)
Wilson B-factor	26.1
*R*_merge_^†^ (%)	0.118 (0.833)
*R*_p.i.m._^+^ (%)	0.069 (0.475)
Half-dataset correlation CC1/2	0.994 (0.815)
Multiplicity	3.9 (4.0)
Total reflections	197358 (14904)
Unique reflections	51674 (5153)
Completeness (%)	97.3 (97.2)
Anomalous completeness (%)	93.2 (93.6)
Anomalous multiplicity	1.9 (2.0)
**Refinement statistics**	
Protein atoms	6073
Zinc ions	4
Water molecules	336
*R*_work_^‡^ (%)	0.194
*R*_free_^§^ (%)	0.237
Root-mean-square deviation (r.m.s.d.) bond lengths (Å)	0.019
R.m.s.d. bond angles (°)	1.93
Average B-factor (Å^2^)	31.0
Protein	
Main-chain (A, B, C, D)	29.1, 30.2, 28.8, 28.3
Side-chain (A, B, C, D)	33.1, 34.8, 32.3, 32.8
Zinc ions (A, B, C, D)	30.5, 24.5, 25.0, 32.1 (occ 1.0)
Water molecules	30.9
**Ramachandran plot**	
Residues in favoured regions (%)	99.7
Residues in allowed regions (%)	0.26
Residues outliers (%)	0.0
PDB (Protein Data Bank) accession code	6FF9

^†^$R_{\mathrm{merge}}=\frac{\sum_{hkl}\sum_{i=1}^{n}\left|I_{i}\left(hkl\right)-\bar{I}\left(hkl\right)\right|}{\sum_{hkl}\sum_{i=1}^{n}I_{i}\left(hkl\right)}$, where I is the observed intensity, and $\bar{I}$ is the statistically weighted average intensity of multiple observations. ^+^ $R_{p.i.m.}=\frac{\sum_{hkl}\sqrt{1/\left(n-1\right)}\sum_{i=1}^{n}\left|I_{i}\left(hkl\right)-\bar{I}\left(hkl\right)\right|}{\sum_{hkl}\sum_{i=1}^{n}I_{i}\left(hkl\right)}$, a redundancy-independent version of $R_{\mathrm{merge}}$. ^‡^ $R_{\mathrm{work}}=\frac{\sum_{hkl}\left|\left|F_{obs}\left(hkl\right)\right|-\left|F_{calc}\left(hkl\right)\right|\right|}{\sum_{hkl}\left|F_{obs}\left(hkl\right)\right|}$, where $\left|F_{calc}\right|$ and $\left|F_{obs}\right|$ are the calculated and observed structure factor amplitudes, respectively. ^§^ *R*_free_ is calculated for a randomly chosen 5% of the reflections.

## Supplementary Materials

Supplementary materials can be found at http://www.mdpi.com/1422-0067/19/4/1184/s1.
