# Supplementary material for: The Crystal Structure of the R280K Mutant of Human p53 Explains the Loss of DNA Binding
Source: Int J Mol Sci. 2018 Apr 13;19(4):1184. doi: 10.3390/ijms19041184 (PMC5979565; doi:10.3390/ijms19041184)
Supplement: Supplementary file 1 [file ijms-19-01184-s001.pdf]

Supplementary Material

“The crystal structure of the R280K mutant of human p53 explains the loss of DNA binding”

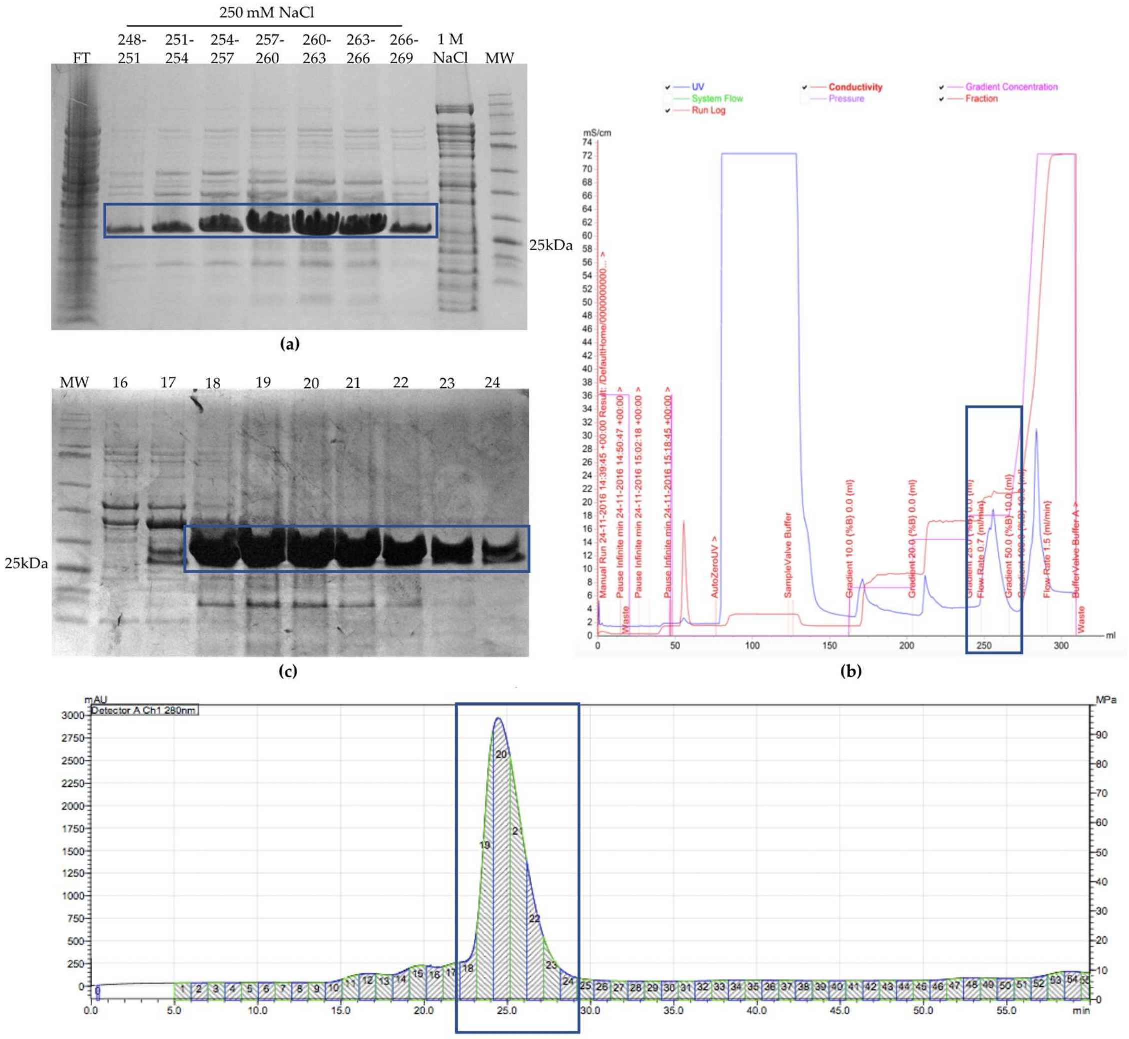

(d)

Figure S1. Purification of mutant p53R280K DBD. Blue squares delimitate the expressed and purified mutant p53R280K DBD protein. **(a)** SDS-page monitoring of mutant p53R280K DBD containing fractions eluted with 250 mM of NaCl (248-269 mL, 3 mL each fraction) after heparin-affinity chromatography. **(b)** Heparin-affinity chromatography profile (blue line - absorbance at 280 nm). **(c)** SDS-page monitoring of mutant p53R280K DBD after gel filtration chromatography. **(d)** Gel filtration chromatography profile (absorbance at 280 nm). FT – flow through; MW – molecular weight.

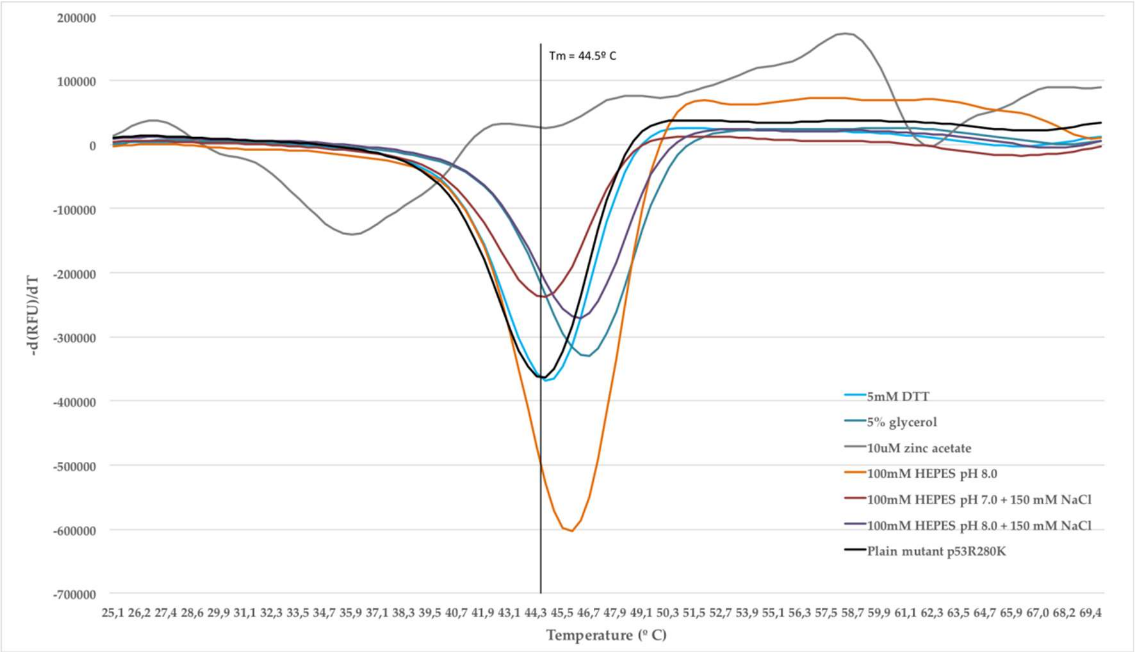

Figure S2. Thermal denaturation of mutant p53R280K DBD obtained using a DSF screening of buffers and additives. The black vertical line indicates the Tm of mutant p53R280K DBD (black curve). The colored curves represent the thermal denaturation of the studied protein in different conditions of buffers and additives that exhibit an increase of Tm, therefore a thermal stability increase. Only the best conditions that enabled to disclose the final buffer composition were represented.

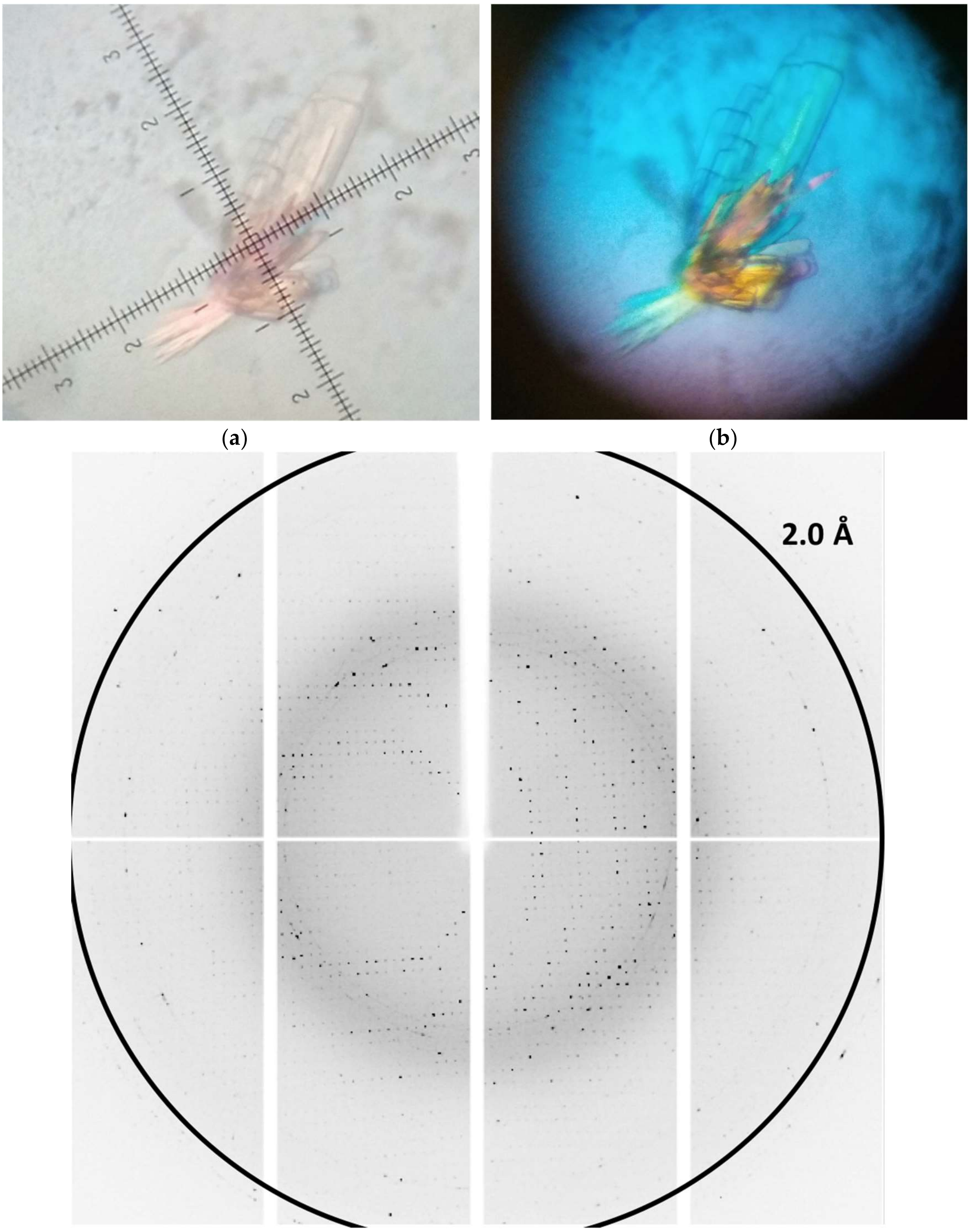

(**a**) (**b**) (**c**)

Figure S3. (a) Agglomerate of plate-shaped crystals of p53R280K grown by the sitting-drop vapour-diffusion method in buffer SB. **(b)** Same crystals viewed under polarized light. The average crystal size is 0.3 x 0.1 mm2. **(c)** X-ray diffraction image of p53R280K. The circle delimitates the high-resolution limit at 2.0 Å. The diffraction image was obtained at 12.81 keV (ID30A-3, ESRF) using a crystal-to-detector distance of 144.8 mm and an oscillation angle of 0.15°.
